# The association of fat and other macronutrients with breast cancer: a case-control study from Greece.

**DOI:** 10.1038/bjc.1994.341

**Published:** 1994-09

**Authors:** K. Katsouyanni, A. Trichopoulou, S. Stuver, Y. Garas, A. Kritselis, G. Kyriakou, M. Stoïkidou, P. Boyle, D. Trichopoulos

**Affiliations:** Department of Hygiene and Epidemiology, University of Athens Medical School, Greece.

## Abstract

The Greek diet is characterized by a high total fat but low saturated fat intake. In a hospital-based case-control study of female breast cancer conducted in Athens (1989-91), 820 patients with confirmed cancer of the breast were compared with 795 orthopaedic patient controls and 753 hospital visitor controls, matched to the cases by age and interviewer. Diet was ascertained through a semiquantitative food frequency questionnaire; macronutrient intakes were estimated from the nutrient content of a selected typical portion size for each specified food item, summed for all items. Logistic regression was used to analyse the data, controlling for demographic and reproductive risk factors for breast cancer as well as for total energy intake and mutual confounding influences among nutrients. There was no significant or suggestive association of total protein, total fat, categories of fat or total carbohydrates with breast cancer risk. Thus, the mutually adjusted relative risk per quintile and (in parenthesis) 95% confidence interval were: for protein, 1.06 (0.94-1.20); saturated fat, 0.99 (0.89-1.11); monounsaturated fat, 0.97 (0.88-1.07), polyunsaturated fat, 1.05 (0.97-1.13); and total carbohydrates, 1.03 (0.94-1.12). In alternative analytical approaches only total protein appeared to be positively associated to the occurrence of breast cancer with some consistency, but the results were far from statistically significant. These findings do not support a role for fat or other energy-generating nutrients in the aetiology of breast cancer.


### The association of fat and other macronutrients with breast cancer: a case-control study from Greece

K. Katsouyanni[1,2], A. Trichopoulou[3], S. Stuver[2], Y. Garas[4], A. Kritselis[5], G. Kyriakou[4], M. Stoïkidou[1], P. Boyle[6] & D. Trichopoulos[2]

[1]*Department of Hygiene and Epidemiology, University of Athens Medical School, Goudi, Athens 115-27, Greece;* [2]*Department of Epidemiology and Center for Cancer Prevention, Harvard School of Public Health, 677 Huntington Avenue, Boston, Massachusetts 02115, USA;* [3]*Department of Nutrition and Biochemistry, Athens School of Public Health, Leoforos Alexandras 196, Athens 115-21, Greece;* [4]*Medical Center Hospital, Athens, Greece;* [5]*Metaxa Cancer Hospital, Piraeus, Greece; and* [6]*Division of Epidemiology and Biostatistics, European Institute of Oncology, Via Ripamonti 332/10, 20141 Milan, Italy.*

**Summary** The Greek diet is characterized by a high total fat but low saturated fat intake. In a hospital-based case-control study of female breast cancer conducted in Athens (1989-91), 820 patients with confirmed cancer of the breast were compared with 795 orthopaedic patient controls and 753 hospital visitor controls, matched to the cases by age and interviewer. Diet was ascertained through a semiquantitative food frequency questionnaire; macronutrient intakes were estimated from the nutrient content of a selected typical portion size for each specified food item, summed for all items. Logistic regression was used to analyse the data, controlling for demographic and reproductive risk factors for breast cancer as well as for total energy intake and mutual confounding influences among nutrients. There was no significant or suggestive association of total protein, total fat, categories of fat or total carbohydrates with breast cancer risk. Thus, the mutually adjusted relative risk per quintile and (in parenthesis) 95% confidence interval were: for protein, 1.06 (0.94-1.20); saturated fat, 0.99 (0.89-1.11); monounsaturated fat, 0.97 (0.88-1.07), polyunsaturated fat, 1.05 (0.97-1.13); and total carbohydrates, 1.03 (0.94-1.12). In alternative analytical approaches only total protein appeared to be positively associated to the occurrence of breast cancer with some consistency, but the results were far from statistically significant. These findings do not support a role for fat or other energy-generating nutrients in the aetiology of breast cancer.

The epidemiology of breast cancer has been studied more than that of any other disease, and several risk factors have been established (Kelsey, 1993). However, a substantial part of the variation of breast cancer occurrence between and within population groups cannot be explained in terms of the identified factors (Seidman *et al.*, 1982; Hsieh *et al.*, 1990). There seems to be little doubt that nutrition plays a central role in the aetiology of this disease, but investigators are divided as to the life stage during which nutritional factors exert their effect. Several scientists believe that qualitative aspects of diet during adult life may be critical determinants of breast cancer risk (Howe *et al.*, 1990; Prentice & Sheppard, 1990), whereas others are of the opinion that the risk for breast cancer is affected by nutrition mainly before or during adolescence (Cole & MacMahon, 1969; Micozzi, 1985; de Waard & Trichopoulos, 1988; Albanes & Winick, 1988; Willett, 1989). The role of adult diet, and in particular fat intake, in the natural history of breast cancer has become the focus of intense debate (Sun, 1988; Michels & Willett, 1991). The collective evidence from case-control studies (La Vecchia *et al.*, 1987; Toniolo *et al.*, 1989; Ewertz & Gill, 1990; Howe *et al.*, 1990; Boyd *et al.*, 1993) suggests that fat intake in adult life may increase the risk for breast cancer, whereas with few exceptions (Knekt *et al.*, 1990; Howe *et al.*, 1991) cohort studies indicate no association between dietary fat in adult life and incidence of breast cancer (Hunter & Willett, 1993). Ecological data (Armstrong & Doll, 1975; Prentice *et al.*, 1988; Prentice & Sheppard, 1990) also support a relationship between dietary fat and breast cancer, but are less powerful than data from retrospective and prospective studies in demonstrating causality. We have examined the association of dietary fat and other macronutrients with breast cancer in Athens through a large case-control study that utilized two independent control series. Average total fat intake is high in Greece, accounting for 42% of total calories, and the variability of fat intake is also considerable (Trichopoulou *et al.*, 1993).

Correspondence: D. Trichopoulos, Department of Epidemiology, Harvard School of Public Health, 677 Huntington Avenue, Boston, MA 02115, USA.
Received 15 November 1993; and in revised form 14 March 1993.

### Subjects and methods

During a 3 year period from January 1989 to December 1991, all newly diagnosed women with breast cancer who were residents of the Greater Athens area (population about 3.5 million) were identified in four major hospitals, representating about 50% of breast cancer cases occurring in this area. The hospitals included in the study were: Athens Medical Center, Elena's Hospital for Women and Agios Savas Cancer Hospital in Athens and Metaxa Cancer Hospital in Piraeus. Eight hundred and seventy-three histologically confirmed cases were identified. It was not possible to interview 53 patients (6%), and 820 were eventually included in the study. Each case was interviewed in the hospital before the first discharge, by specially trained interviewers. The interview lasted 45-65 min and was based on a structured questionnaire.

For each case, two controls were to be selected: one from among hospital visitors (excluding first-degree relatives and women who have had breast cancer) in the same hospital; the other, an orthopaedic patient in the major accident hospital of Athens (for breast cancer cases who were residents of Athens and surroundings) or Piraeus (for cases who were residents of Piraeus and surroundings). Each control had to be ± 5 years of age with respect to the index case, and all controls were residents of the same area. Eight hundred and thirty eligible hospital controls and 808 eligible visitor controls were identified. We were not able to interview 35 (4%) hospital controls and 55 (7%) visitor controls. Among the hospital controls, 342 (43%) had fractures, 223 (28%) had arthroplasty and the remaining 230 (29%) had other orthopaedic conditions. In none of these middle-aged or older women with fractures was alcohol intake reported as a contributing factor in the occurrence of the incident that had caused the fracture. All controls were interviewed in the hospital using the same questionnaire as for the cases. Every case-control triplet was interviewed by the same interviewer.

The questionnaire covered demographic, socioeconomic, reproductive and biomedical variables as well as including a semiquantitative food frequency section. Specifically, all subjects were asked to indicate the average frequency of consumption, over a period of 1 year before onset of the present disease (or before the interview for visitor controls), of 115 food items or beverage categories per month, per week or per day. For analysis, the frequency of consumption of different food items was quantified approximately in terms of the number of times per month the food was consumed, as done by Graham *et al.* (1978) and Katsouyanni *et al.* (1991*a*). Thus daily consumption was multiplied by 30 and weekly consumption by 4, while a value of 0 was assigned to food items rarely or never consumed.

Nutrient intakes for individuals were estimated by multiplying the nutrient contents of a selected typical portion, for each specified food item, by the frequency that the food item was eaten per month and adding these estimates for all food items. Food consumption data were based on a nutrient database developed in Greece by the Department of Nutrition and Biochemistry, Athens School of Public Health (Trichopoulou, 1992). The portion size estimation was based on the results from previous validation studies (Katsouyanni *et al.*, 1991*b*; Gnardellis *et al.*, 1994), and the nutrient content was calculated on the basis of Greek recipes (Trichopoulou, 1992). The macronutrient intakes used were: protein (g), total fat (g), saturated, monounsaturated and polyunsaturated fat (g) and carbohydrates (g), as well as total energy (kcal). In order to investigate the relation of the estimated nutrient intakes to breast cancer risk, a preliminary analysis was undertaken based on the comparison of the frequency distribution of cases and controls by marginal quintiles of individual nutritional factors. No differences have been found among methods for quantile classification of exposure levels in case–control studies; thus, use of the marginal frequencies was based on practical advantages (Hsieh *et al.*, 1991). Since most nutrients are positively correlated with total energy (Willett & Stampfer, 1986), calorie adjustment was utilized. Nutrient intakes were alternately or simultaneously used as independent variables.

Controls were paired to cases in order to control for patient origin and interviewer identity. However, only 680 complete triplets were available, and for these conditional and unconditional logistic regression (controlling for the matching factors) generated identical results. Therefore all cases and controls were utilised, and the data were modelled through unconditional logistic regression using the SPSS (1980) statistical package. Comparison of breast cancer cases with either control series generated similar results, as will be seen in the Results section. Thus, for most analyses, the two control series were combined in order to increase the precision of the effect estimates. Furthermore, cross-tabulated analysis by hospital did not reveal any systematic or striking differences.

Since demographic and reproductive risk factors for breast cancer are well established, a core model was used that included: age (years), place of birth (urban, rural), Quetelet index (kg $m^{-2}$), parity (parous, nulliparous), age at first pregnancy (years; among parous women), age at menarche (years) and menopausal status (post-menopausal, premenopausal). Exogenous oestrogens are rarely used in Greece, and there was no reason to control for use of oral contraceptives and menopausal oestrogens (among cases, 40 had ever used oral contraceptives and 57 menopausal oestrogens; among all controls, the corresponding numbers were 66 and 98).

### Results

Table I shows the distribution of cases and controls by age, place of birth, Quetelet index, parity, age at first birth, age at menarche and menopausal status, i.e. the variables that were included in the core model. Table II shows results from the multiple logistic regression model that included these variables. The results are compatible with the established risk profile of breast cancer.

Table III shows the distribution of cases and controls by marginal quintiles of total energy intake and energy-generating macronutrients as well as age-adjusted $\chi$ values for linear trend. It appears that cases have a tendency to over-report, which is accounted for in subsequent nutritional analyses by controlling for energy intake. There seems to be little evidence that any macronutrient is specifically and disproportionately associated with breast cancer risk. However, these data are inherently confounded and therefore not directly interpretable.

Table IV shows the nutrient-specific associations with breast cancer risk controlling for energy intake and the variables in the core model (Table II), but without mutual adjustment. The regression coefficients refer to a one quintile increase in the relevant nutrient. There is no evidence for a substantial or significant association of any macronutrient with breast cancer risk. The corresponding associations after controlling for mutual confounding among macronutrients as well as for the core variables are shown in Table V. Total fats are not included to avoid collinearity problems. Again, no energy-generating nutrient appears to be associated with breast cancer risk.

In Table VI adjustment for energy intake is done with the Willett–Stampfer (Willett & Stampfer, 1986) method, which uses nutrient residuals from the energy-predicted estimates for the corresponding nutrients. Furthermore, in Table VI, breast cancer cases are compared with each control series as well as with both control series combined. The purpose of this table is to explore whether alternative analytical approaches might generate different impressions. There is no such evidence. The results confirm that there are no significant or substantial associations between any of the macronutrient groups and breast cancer risk. Only protein appears to be positively associated with the risk for breast cancer in both Tables V and VI, but the results are not statistically significant. Modelling these data without mutual adjustment among macronutrients led to essentially the same conclusions. In addition, separate use of hospital controls with fractures and hospital controls with arthroplasty or other conditions did not generate inconsistent results.

**Table I** Distribution of 820 cases of breast cancer and 1,548 controls[a] by age, place of birth, Quetelet index, parity, age at first birth, age at menarche and menopausal status

| | *Cases* | | *Controls* | |
|---|---|---|---|---|
| Age (years) | 56.4 | (0.43)[b] | 54.4 | (0.32) |
| Place of birth | | | | |
| Urban | 620 | (75.7) | 1106 | (71.6) |
| Rural | 199 | (24.3) | 439 | (28.4) |
| Quetelet index (kg $m^{-2}$) | 26.6 | (1.02) | 25.9 | (0.75) |
| Ever pregnant | | | | |
| Yes | 657 | (80.2) | 1164 | (75.2) |
| No | 162 | (19.8) | 384 | (24.8) |
| Age at first birth (years) | 26.4 | (0.21) | 25.9 | (0.16) |
| Age at menarche (years) | 12.9 | (0.06) | 13.1 | (0.04) |
| Menopausal status | | | | |
| Post-menopausal | 550 | (67.1) | 1041 | (67.3) |
| Premenopausal | 270 | (32.9) | 505 | (32.7) |

[a]Non-additivity is accounted for by a few missing values.

[b]In parenthesis: for quantitative variables, standard errors; for qualitative variables, percentages.

**Table II** Multiple logistic regression-derived, mutually adjusted relative risks for breast cancer, according to selected demographic and reproductive variables

| *Variable* | *Groups or units* | *Relative risk* | P-*value (two tailed)* |
|---|---|---|---|
| Age | 1 year | 1.03 | $<10^{-3}$ |
| Birth place | Rural *vs* urban | 0.84 | 0.10 |
| Quetelet index | 1 kg $m^{-2}$ | 0.99 | 0.78 |
| Ever pregnant | Yes *vs* no | 0.79 | 0.38 |
| Age at first birth | 1 year | 1.02 | 0.07 |
| Age at menarche | 1 year | 0.94 | 0.03 |
| Menopausal status | Post *vs* pre | 0.56 | $<10^{-3}$ |

**Table III** Distribution of 820 cases and 1,548 controls by marginal quintiles of total energy and major macronutrient intake[a]

| | | Quintile[b] 1 | 2 | 3 | 4 | 5 | Total | χ, linear trend[c] | P-value |
|---|---|---|---|---|---|---|---|---|---|
| Total energy (kcal) | Cases | 148 | 166 | 166 | 160 | 176 | 816 | 2.05 | 0.04 |
| | Controls | 319 | 307 | 308 | 313 | 298 | 1,545 | | |
| Protein (g) | Cases | 145 | 160 | 184 | 142 | 188 | 819 | 2.66 | 0.01 |
| | Controls | 329 | 312 | 290 | 331 | 285 | 1,547 | | |
| Total fat (g) | Cases | 157 | 164 | 175 | 153 | 170 | 819 | 1.13 | 0.26 |
| | Controls | 315 | 310 | 300 | 319 | 303 | 1,547 | | |
| Saturated fat (g) | Cases | 154 | 157 | 176 | 155 | 177 | 819 | 1.85 | 0.06 |
| | Controls | 319 | 316 | 298 | 318 | 296 | 1,547 | | |
| Monounsaturated fat (g) | Cases | 153 | 165 | 172 | 156 | 173 | 819 | 1.66 | 0.10 |
| | Controls | 320 | 309 | 301 | 318 | 299 | 1,547 | | |
| Polyunsaturated fat (g) | Cases | 164 | 149 | 161 | 170 | 175 | 819 | 2.05 | 0.04 |
| | Controls | 309 | 324 | 313 | 304 | 297 | 1,547 | | |
| Carbohydrates (g) | Cases | 143 | 160 | 181 | 173 | 162 | 819 | 2.02 | 0.04 |
| | Controls | 330 | 313 | 293 | 300 | 311 | 1,547 | | |

[a]There are a few missing values. [b]The quintile ranges (per day) are as follows: total energy (kcal), ≤1,521.8, 1,521.9–1,752.8, 1,752.9–1,980.0, 1,980.1–2,266.0, ≥2,266.1; protein (g), ≤57.9, 58.0–67.2, 67.3–75.9, 76.0–89.4, ≥89.5; total fat (g), ≤79.9, 80.0–91.2, 91.3–101.9, 102.0–119.2, ≥119.3; saturated fat (g), ≤27.0, 27.1–32.4, 32.5–37.4, 37.5–44.7, ≥44.8; monounsaturated fat (g), ≤40.1, 40.2–44.7, 44.8–50.3, 50.4–59.0, ≥59.1; polyunsaturated fat (g), ≤8.7, 8.8–10.6, 10.7–12.6, 12.7–16.1, ≥16.2; carbohydrates (g), ≤137.2, 137.3–173.1, 173.2–203.5, 203.6–243.0, ≥243.1. [c]χ values are age adjusted.

**Table IV** Multiple logistic regression-derived coefficients for the major macronutrients[a]

| Nutrient (g) | b[b] | s.e.[c] | P-value | Relative risk | 95% confidence interval |
|---|---|---|---|---|---|
| Protein | 0.0703 | 0.0618 | 0.26 | 1.07 | 0.95–1.21 |
| Total fat | −0.0585 | 0.0533 | 0.27 | 0.94 | 0.85–1.05 |
| Saturated fat | −0.0008 | 0.0469 | 0.99 | 1.00 | 0.91–1.10 |
| Monounsaturated fat | −0.0207 | 0.0467 | 0.66 | 0.98 | 0.89–1.07 |
| Polyunsaturated fat | 0.0404 | 0.0388 | 0.30 | 1.04 | 0.97–1.12 |
| Carbohydrates | 0.0508 | 0.0553 | 0.36 | 1.05 | 0.95–1.17 |

[a]Coefficients in this table are not mutually adjusted, but they are adjusted for age, place of birth, parity, age at first pregnancy, age at menarche, menopausal status, Quetelet index and total energy intake. [b]Coefficient (b) from logistic regression model, per quintile. [c]Standard error (s.e.) around the coefficient.

**Table V** Multiple logistic regression-derived, mutually adjusted coefficients for the major macronutrients[a]

| Variable | b[b] | s.e.[c] | P-value | Relative risk | 95% confidence interval |
|---|---|---|---|---|---|
| Protein | 0.0589 | 0.0625 | 0.35 | 1.06 | 0.94–1.20 |
| Saturated fat | −0.0097 | 0.0569 | 0.86 | 0.99 | 0.89–1.11 |
| Monounsaturatedfat | −0.0297 | 0.0479 | 0.53 | 0.97 | 0.88–1.07 |
| Polyunsaturated fat | 0.0486 | 0.0390 | 0.21 | 1.05 | 0.97–1.13 |
| Carbohydrates | 0.0286 | 0.0437 | 0.51 | 1.03 | 0.94–1.12 |

[a]Controlling also for age, place of birth, parity, age at first pregnancy, age at menarche, menopausal status, Quetelet index and total energy intake. [b]Coefficient (b) from logistic regression model, per quintile. [c]Standard error (s.e.) around the coefficient.

**Table VI** Multiple logistic regression-derived, mutually adjusted coefficients for the major macronutrients, controlling for the core variables and for energy intake with the Willett–Stampfer approach (Willett & Stampfer, 1986) and considering each control series separately as well as together

| Variable | Hospital controls b[b] | s.e.[c] | P-value | Visitor controls b | s.e. | P-value | Combined control series[a] b | s.e. | P-value |
|---|---|---|---|---|---|---|---|---|---|
| Saturated fat | 0.0068 | 0.0457 | 0.88 | 0.0456 | 0.0637 | 0.47 | −0.0311 | 0.0527 | 0.56 |
| Monounsaturated fat | 0.0339 | 0.0538 | 0.53 | −0.0025 | 0.0564 | 0.96 | −0.0111 | 0.0475 | 0.82 |
| Polyunsaturated fat | 0.0508 | 0.0419 | 0.23 | −0.0125 | 0.0433 | 0.77 | −0.0010 | 0.0366 | 0.98 |
| Carbohydrates | 0.1029 | 0.0704 | 0.14 | −0.0749 | 0.0841 | 0.37 | −0.0627 | 0.0697 | 0.37 |
| Protein[d] | 0.0758 | 0.0417 | 0.07 | 0.0166 | 0.0425 | 0.70 | 0.0534 | 0.0363 | 0.14 |

[a]Calculation of residuals from a combined database and a different constellation of confounding factors when the control series are combined explain why the 'combined' regression coefficients deviate from the means of the control series-specific regression coefficients. [b]Coefficient (b) from logistic regression model, per quintile. [c]Standard error (s.e.) around the coefficient. [d]To avoid collinearity, protein estimates were derived from a model in which protein was substituted for carbohydrates.

### Discussion

This study is large, which reduces the role of chance variation to acceptable levels. Adjustment for known demographic and reproductive risk factors for breast cancer controlled for this source of confounding, whereas mutual confounding among macronutrients was accounted for to the extent permitted by the inherently limited validity of dietary ascertainment in any investigation in nutritional epidemiology (Tzonou *et al.*, 1986). As in any case–control study, the dominant concerns are selection and information bias. Low rates of non-response among all three series and similarity of findings when cases were compared with either of the two control series suggest that overt selection bias was not operating in the investigation.

Women with breast cancer are likely to report more completely or over-report their dietary intakes in comparison with women in the control series. This was evident in the present study (Table III) as well as in many earlier case–control investigations (Toniolo *et al.*, 1989; review by Hunter & Willett, 1993). However, over-reporting, or more complete reporting, by cases is likely to cover foods in general rather than specific food items with particularly high or low contents of specific nutrients. Few persons, and certainly very few Greek women, have specific ideas, let alone beliefs, about the nutritional aetiology of breast cancer *and* sufficient knowledge about the nutritional content of the various food items. When over-reporting is general (Table III), adjustment for energy intake (Willett & Stampfer, 1986; Willett, 1990) eliminates the consequences of over-reporting information bias (Trichopoulos *et al.*, 1991). Indeed, in a comparison of prospective and retrospective assessment of diet in the aetiology of breast cancer (Giovannucci *et al.*, 1993), retrospective (case–control) data on fat consumption with over-reporting by cases generated a null result after adjustment for energy intake ($P \sim 0.78$). This appears to be biologically more plausible than the finding of the prospective component of the investigation, suggesting an almost significant *inverse* association between fat intake and breast cancer (Table 4 of Giovannucci *et al.*, 1993). Furthermore, in the Giovannucci *et al.* (1993) study, retrospective data were available for only 77% of the study subjects included in the prospective component. Information bias does not appear to be an intractable problem in adequately planned and carefully analysed case–control studies of diet and cancer.

The hypothesis linking dietary fat to breast cancer risk is biologically credible. Animal data are widely considered as compatible with a promoting or growth-enhancing effect of fat intake on the occurrence of breast cancer (Tannenbaum, 1942; National Academy of Sciences, 1982; Birt, 1986; National Research Council, 1989; Freedman *et al.*, 1990; World Health Organization, 1990). Ecological correlations and time trends are frequently viewed as supportive of a positive association between fat intake and breast cancer risk (Prentice *et al.*, 1988; Prentice & Sheppard, 1990). Dietary fat also has been shown in small human studies to be correlated with oestrogen levels (Goldin *et al.*, 1986; Rose *et al.*, 1987; Boyar *et al.*, 1988; Adlercreutz, 1990; Bennett & Ingram, 1990; Prentice *et al.*, 1990), and several well-conducted case–control studies have suggested that there may indeed be a weak positive association between fat intake and breast cancer (La Vecchia *et al.*, 1987; Toniolo *et al.*, 1989; Howe *et al.*, 1990). However, the animal studies often have been confounded by total energy intake; the ecological studies are susceptible to extensive confounding; the metabolic studies have for the most part lacked appropriate control groups; and selection bias could not be excluded in some case–control studies (Willett & Stampfer, 1990). By contrast, the results of the largest cohort study undertaken until now, the Nurses' Health Study (Willett *et al.*, 1992), do not support the existence of a positive association, and the collective evidence from other smaller cohort studies on fat intake and breast cancer risk is compatible with the absence of an association or a minimal increase of risk (Hunter & Willett, 1993).

Some scientists have argued that the lack of an association in the Nurses' Health Study may be due to the limited range of variation in that population (Prentice *et al.*, 1988; Toniolo *et al.*, 1989; Prentice *et al.*, 1990). This is an unlikely explanation, since the post estimates of regression coefficients do not depend on the range of variation and the power implications, under realistic conditions, are generally small. In any case, the range of variation of fat intake in the present study was wide. With total fat expressed as a percentage of calories, these Greek women had a mean contribution to total energy from fat of 60% in the highest quintile and of 36% in the lowest quintile. The corresponding figures in an Italian study that reported a significant association of fat intake with breast cancer risk were 46% and 26% (Toniolo *et al.*, 1989).

The present study was large and, in all evidence, adequately controlled with two independent control series. Variation of fat intake was substantial in the study base, the dietary questionnaire was close to exhaustive, and the analysis adequately accounted for confounding by energy intake, for mutual confounding among nutrients and for bias due to case over-reporting. The results do not support hypotheses invoking fat, saturated fat, other fats or other macronutrients in the adult diet as important risk factors for breast cancer. It should be added that in earlier case–control studies conducted in Greece, total fat and animal fat-containing foods (meat and meat products) have been found to significantly increase the risk of coronary heart disease (Tzonou *et al.*, 1993) and cancer of the large bowel (Manousos *et al.*, 1983; Trichopoulou *et al.*, 1992), whereas in an earlier, independent, smaller case–control study of diet and breast cancer no association with fat was noted (Katsouyanni *et al.*, 1986; Katsouyanni *et al.*, 1988). The relatively low mortality from breast cancer in Greece (about 65% of the corresponding mortality in the USA) is in iself an argument against an important role of dietary fat in the genesis of breast cancer, since fat intake, mostly in the form of olive oil, is very high in Greece (about 42% of total energy intake) (Trichopoulou *et al.*, 1993).

There was no evidence in the present study for a substantial, significant or differential effect of any fat category, total carbohydrates or total protein. Nevertheless, protein was the only macronutrient for which the evidence was not altogether reassuring (Tables IV and V). Protein intake has not been generally considered an important risk factor in breast carcinogenesis. Given the constraints of the study design, it was not possible to examine directly the hypotheses that intake of energy or particular macronutrients (including fats or proteins) in early life stages may be important determinants of breast cancer risk.

This study was supported through grants from the Europe Against Cancer Program of the European Community and from the Central Scientific Health Council of the Greek Ministry of Health (ΚΕΣΥ). In addition, the study was part of an international collaborative programme in breast cancer at the European Institute on Oncology, which is supported by the Associazone Italiana per la Ricerca del Cancro (Italian Association for Cancer Research). Dr Stuver received support from a National Research Service Award in Cancer Epidemiology from the National Cancer Institute, USA (5 T32 CA09001). The collaboration of many cancer surgeons and physicians in patient accrual is also gratefully acknowledged.

### References

ADLERCREUTZ, H. (1990). Western diet and Western diseases: some hormonal and biochemical mechanisms and associations. *Scand. J. Clin. Lab. Invest.*, **50** (Suppl. 201), 3–23.

ALBANES, D. & WINICK, M. (1988). Are cell number and cell proliferation risk factors for cancer? *J. Natl Cancer Inst.*, **80**, 772–775.

ARMSTRONG, B. & DOLL, R. (1975). Environmental factors and cancer incidence and mortality in different countries, with special reference to dietary practices. *Int. J. Cancer*, **15**, 617–631.

BENNETT, F.C. & INGRAM, D.M. (1990). Diet and female sex hormone concentrations: an intervention study for the type of fat consumed. *Am. J. Clin. Nutr.*, **52**, 808–812.

BIRT, D.F. (1986). Dietary fat and experimental carcinogenesis: a summary of recent in vivo studies. *Adv. Exp. Med. Biol.*, **206**, 69–84.

BOYAR, A.P., ROSE, D.P., LOUGHRIDGE, J.R., ENGLE, A., POLGI, A., LAASKO, K., KINNE, D. & WYNDER, E.L. (1988). Response to a diet low in total fat in women with postmenopausal breast cancer. *Nutr. Cancer*, **11**, 93–99.

BOYD, N.F., MARTIN, L.J., NOFFEL, M., LOCKWOOD, G.A. & TRICHLER, D.L. (1993). A meta-analysis of studies of dietary fat and breast cancer. *Br. J. Cancer*, **68**, 627–636.

COLE, P. & MACMAHON, B. (1969). Oestrogen fractions during early reproductive life in the aetiology of breast cancer. *Lancet*, **i**, 604–606.

DE WAARD, F. & TRICHOPOULOS, D. (1988). A unifying concept of the aetiology of breast cancer. *Int. J. Cancer*, **41**, 666–669.

EWERTZ, M. & GILL, C. (1990). Dietary factors and breast cancer risk in Denmark. *Int. J. Cancer*, **46**, 779–784.

FREEDMAN, L.S., CLIFFORD, C. & MESSINA, M. (1990). Analysis of dietary fat, calories, body weight, and the development of mammary tumors in rats and mice: a review. *Cancer Res.*, **50**, 5710–5719.

GIOVANNUCCI, E., STAMPFER, M.J., COLDITZ, G.A., MANSON, J.E., ROSNER, B.A., LONGNECKER, M., SPEIZER, F.E. & WILLETT, W.C. (1993). A comparison of prospective and retrospective assessments of diet in the study of breast cancer. *Am. J. Epidemiol.*, **137**, 502–511.

GNARDELLIS, C., TRICHOPOULOU, A., KATSOUYANNI, K., POLYCHRONOPOULOS, E., RIMM, E.B. & TRICHOPOULOS, D. (1994). Reproducibility and validity of an extensive semi-quantitative food frequency questionnaire among Greek school teachers. *Epidemiology*, (in press).

GOLDIN, B.R., ADLERCREUTZ, H., GORBACH, S.L., WOODS, M.N., DWYER, J.T., CONLON, T., BOHN, E. & GERSCHOFF, S.N. (1986). The relationship between estrogen levels and diets of Caucasian American and Oriental immigrant women. *Am. J. Clin. Nutr.*, **44**, 945–953.

GRAHAM, S., DAYAL, H., SWANSON, M., MITTELMAN, A. & WILKINSON, G. (1978). Diet in the epidemiology of cancer of the colon and rectum. *J. Natl Cancer Inst.*, **61**, 709–714.

HOWE, G.R., HIROHATA, T., HISLOP, T.G., ICOVICH, J.M., YUAN, J.M., KATSOUYANNI, K., LUBIN, F., MARUBINI, E., MODAN, B., ROHAN, T., TONIOLO, P. & SHUNZHANG, Y. (1990). Dietary factors and risk of breast cancer: combined analysis of 12 case–control studies. *J Natl Cancer Inst.*, **82**, 561–569.

HOWE, G.R., FRIEDENREICH, C.M., JAIN, M. & MILLER, A.B. (1991). A cohort study of fat intake and risk of breast cancer. *J. Natl Cancer Inst.*, **83**, 336–340.

HSIEH, C.-C., TRICHOPOULOS, D., KATSOUYANNI, K. & YUASA, S. (1990). Age at menarche, age at menopause, height and obesity as risk factors for breast cancer: associations and interactions in an international case–control study. *Int. J. Cancer*, **46**, 796–800.

HSIEH, C.-C., MAISONNEUVE, P., BOYLE, P., MACFARLANE, G.J. & ROBERTSON, C. (1991). Analysis of quantitative data by quantiles in epidemiologic studies: classification according to cases, noncases, or all subjects? *Epidemiology*, **2**, 137–140.

HUNTER, D.J. & WILLETT, W.C. (1993). Diet, body size, and breast cancer. *Epidemiol. Rev.*, **15**, 110–132.

KATSOUYANNI, K., TRICHOPOULOS, D., BOYLE, P., XIROUCHAKI, E., TRICHOPOULOU, A., LISSEOS, B., VASILAROS, S. & MACMAHON, B. (1986). Diet and breast cancer: a case–control study in Greece. *Int. J. Cancer*, **38**, 815–820.

KATSOUYANNI, K., WILLETT, W., TRICHOPOULOS, D., BOYLE, P., TRICHOPOULOU, A., VASILAROS, S., PAPADIAMANTIS, S. & MACMAHON, B. (1988). Risk of breast cancer among Greek women in relation to nutrient intake. *Cancer*, **61**, 181–185.

KATSOUYANNI, K., SKALKIDIS, Y., PETRIDOU, E., POLYCHRONOPOULOU-TRICHOPOULOU, A., WILLETT, W. & TRICHOPOULOS, D. (1991*a*). Diet and peripheral arterial occlusive disease: the role of poly-, mono-, and saturated fatty acids. *Am. J. Epidemiol.*, **133**, 24–31.

KATSOUYANNI, K., TRICHOPOULOU, A., TRICHOPOULOS, D. & WILLETT, W. (1991*b*). *Dietary Variability in Greece. A Report to the Secretaria of Research and Technology*, pp. 1–64. Ministry of Industry, Research and Technology: Athens.

KELSEY, J.L. (ed.) (1993). *Breast Cancer, Epidemiologic Reviews*, Vol. 15. Johns Hopkins University School of Hygiene and Public Health: Baltimore, MD.

KNEKT, P., ALBANES, D., SEPPANEN, R., AROMAA, A., JARVINEN, R., HYVONEN, L., TEPPO, L. & PUKKALA, E. (1990). Dietary fat and risk of breast cancer. *Am. J. Clin Nutr.*, **52**, 903–908.

LA VECCHIA, C., DECARLI, A., FRANCESCHI, S., GENTILE, A., NEGRI, E. & PARAZZINI, F. (1987). Dietary factors and the risk of breast cancer. *Nutr. Cancer*, **10**, 205–214.

MANOUSOS, O., DAY, N., TRICHOPOULOS, D., GEROVASSILIS, F., TZONOU, A. & POLYCHRONOPOULOU, A. (1983). Diet and colorectal cancer: a case–control study in Greece. *Int. J. Cancer*, **32**, 1–5.

MICHELS, K.B. & WILLETT, W.C. (1991). The women's health initiative: daughter of politics or science? In *Cancer Prevention*, Devita Jr, V.T. Hellman, S. & Rosenberg, S.A. (eds) pp. 1–11. J.B. Lippincott: Philadelphia.

MICOZZI, M.S. (1985). Nutrition, body size, and breast cancer. *Yearbook of Physical Anthropology*, **28**, 175–206.

NATIONAL ACADEMY OF SCIENCES, COMMITTEE ON DIET, NUTRITION AND CANCER (1982). *Diet, Nutrition, and Cancer*. National Academy Press: Washington, DC.

NATIONAL RESEARCH COUNCIL (US), COMMITTEE ON DIET AND HEALTH (1989). *Diet and Health. Implications for Reducing Chronic Disease Risk*. National Academy Press: Washington, DC.

PRENTICE, R.L. & SHEPPARD, L. (1990). Dietary fat and cancer: consistency of the epidemiology data, and disease prevention that may follow from a practical reduction in fat consumption. *Cancer Causes Control*, **1**, 81–97.

PRENTICE, R.L., KAKAR, F., HURSTING, S., SHEPPARD, L., KLEIN, R. & KUSHI, L.H. (1988). Aspects of the rationale for the Women's Health Trial. *J. Natl Cancer Inst.*, **80**, 802–814.

PRENTICE, R.L., THOMPSON, D., CLIFFORD, C., GORBACH, S., GOLDIN, B. & BYAR, D. (1990). Dietary fat reduction and plasma estradiol concentration in healthy postmenopausal women. *J. Natl Cancer Inst.*, **82**, 129–133.

ROSE, D.P., BOYAR, A.P., COHEN, C. & STRONG, L.E. (1987). Effect of a low-fat diet on hormone levels in women with cystic breast disease. I. Serum steroids and gonadotropins. *J. Natl Cancer Inst.*, **78**, 623–626.

SEIDMAN, H., STELLMAN, D.S. & MUSHINSKI, H.M. (1982). A different perspective on breast cancer risk factors: some implications of the nonattributable risk. *CA–Cancer J. Clin.*, **32**, 301–313.

SPSS (1980). *SPSS Users' Guide*. SPSS: Chicago, IL.

SUN, M. (1988). Debate rages over breast cancer study. *Science*, **239**, 17–18.

TANNENBAUM, A. (1942). The genesis and growth of tumours. III. Effects of a high fat diet. *Cancer Res.*, **2**, 468–475.

TONIOLO, P., RIBOLI, E., PROTTA, M. & CAPPA, A.P.M. (1989). Calorie-providing nutrients and risk of breast cancer. *J. Natl Cancer Inst.*, **81**, 278–286.

TRICHOPOULOS, D., TZONOU, A., KATSOUYANNI, K. & TRICHOPOULOU, A. (1991). Diet and cancer: the role of case-control studies. *Ann. Nutr. Metab.*, **35** (Suppl. 1), 89–92.

TRICHOPOULOU, A. (1992). *Composition of Greek Foods and Dishes* (in Greek and English). Athens School of Public Health: Athens.

TRICHOPOULOU, A., TZONOU, A., HSIEH, C.-C., TOUPADAKI, N., MANOUSOS, O. & TRICHOPOULOS, D. (1992). High protein, saturated fat and cholesterol diet, and low levels of serum lipids in colorectal cancer. *Int. J. Cancer*, **51**, 386–389.

TRICHOPOULOU, A., TOUPADAKI, N., TZONOU, A., KATSOUYANNI, K., MANOUSOS, O., KADA, E. & TRICHOPOULOS, D. (1993). The macronutrient composition of the Greek diet: estimates derived from six case-control studies. *Eur. J. Clin. Nutr.*, **47**, 549–558.

TZONOU, A., KALDOR, J., SMITH, P., DAY, N. & TRICHOPOULOS, D. (1986). Misclassification in case–control studies with two dichotomous risk factors. *Rev. Epidem. Santé Publ.*, **34**, 10–17.

TZONOU, A., KALANDIDI, A., TRICHOPOULOU, A., HSIEH, C.-C., TOUPADAKI, N., WILLETT, W. & TRICHOPOULOS, D. (1993). Diet and coronary heart disease: a case–control study in Athens, Greece. *Epidemiology*, **4**, 511–516.

WILLETT, W. (1989). The search for the causes of breast and colon cancer. *Nature*, **338**, 389–394.

WILLETT, W. (1990). *Nutritional Epidemiology*, Monographs in Epidemiology and Biostatistics. Vol. 15. Oxford University Press: New York.

WILLETT, W. & STAMPFER, M.J. (1986). Total energy intake: implications for epidemiologic analyses. *Am. J. Epidermiol.*, **124**, 17–27.

WILLETT, W.C. & STAMPFER, M.J. (1990). Dietary fat and cancer: another view. *Cancer Causes Control*, **1**, 103–109.

WILLETT, W.C., HUNTER, D.J., STAMPFER, M.J., COLDITZ, G., MANSON, J.E., SPIEGELMAN, D., ROSNER, B., HENNEKENS, C.H. & SPEIZER, F.E. (1992). Dietary fat and fiber in relation to risk of breast cancer. *JAMA*, **268**, 2037–2044.

WORLD HEALTH ORGANIZATION, STUDY GROUP ON DIET, NUTRITION AND PREVENTION OF NONCOMMUNICABLE DISEASES (1990). *Diet, Nutrition and the Prevention of Chronic Diseases: Report of a WHO Study Group*, WHO Technical Report Series No. 797. World Health Organization: Geneva.


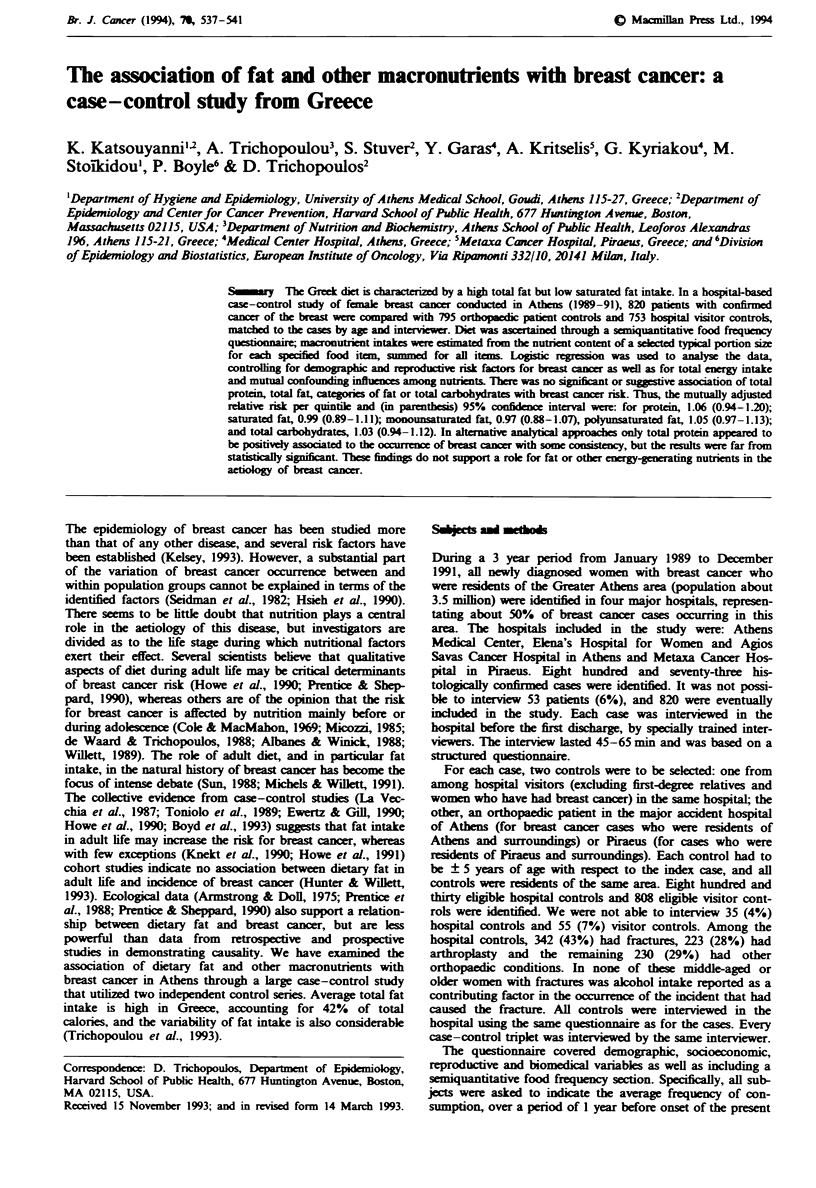

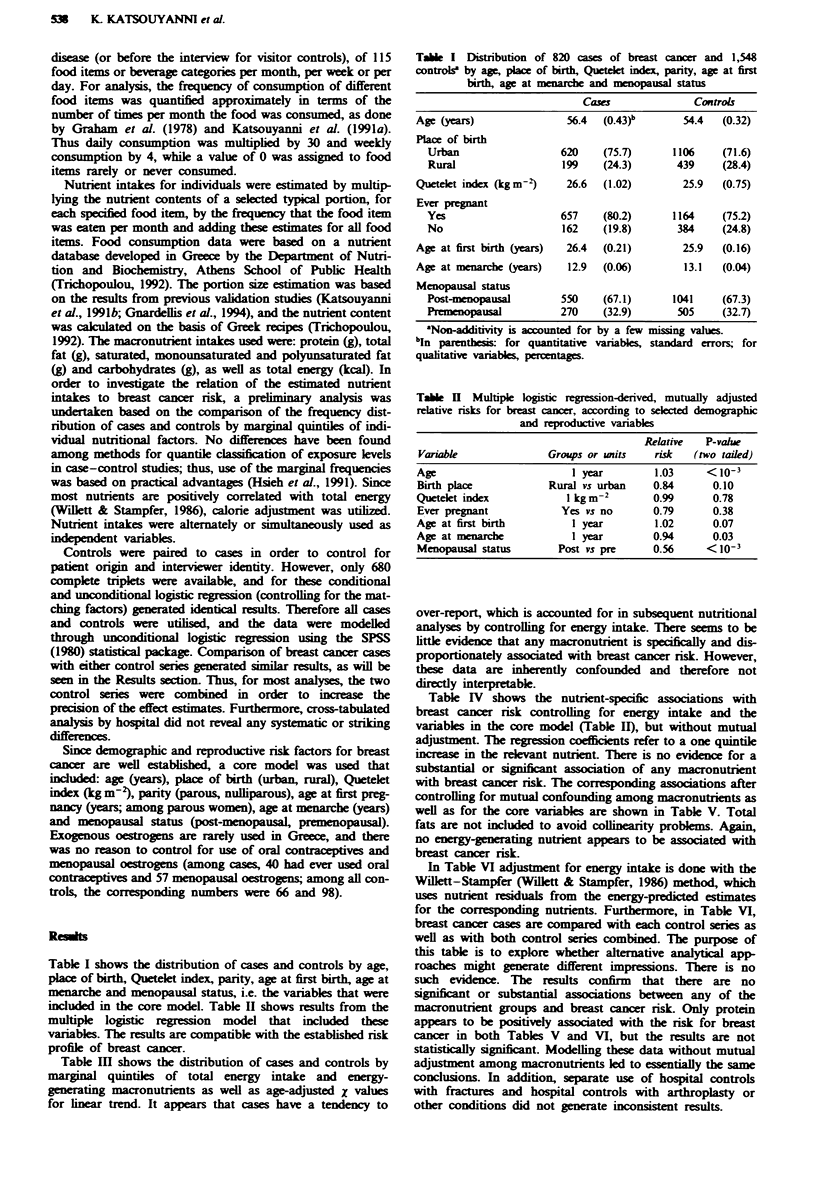

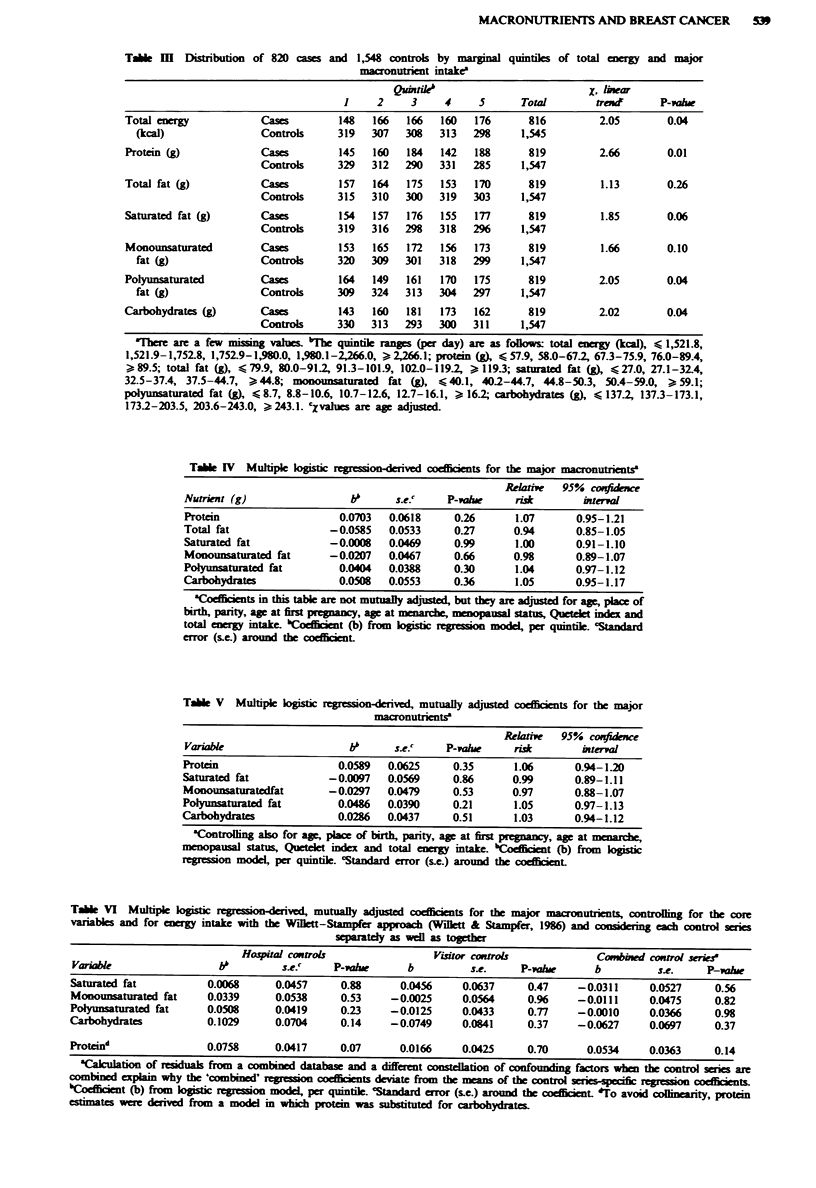

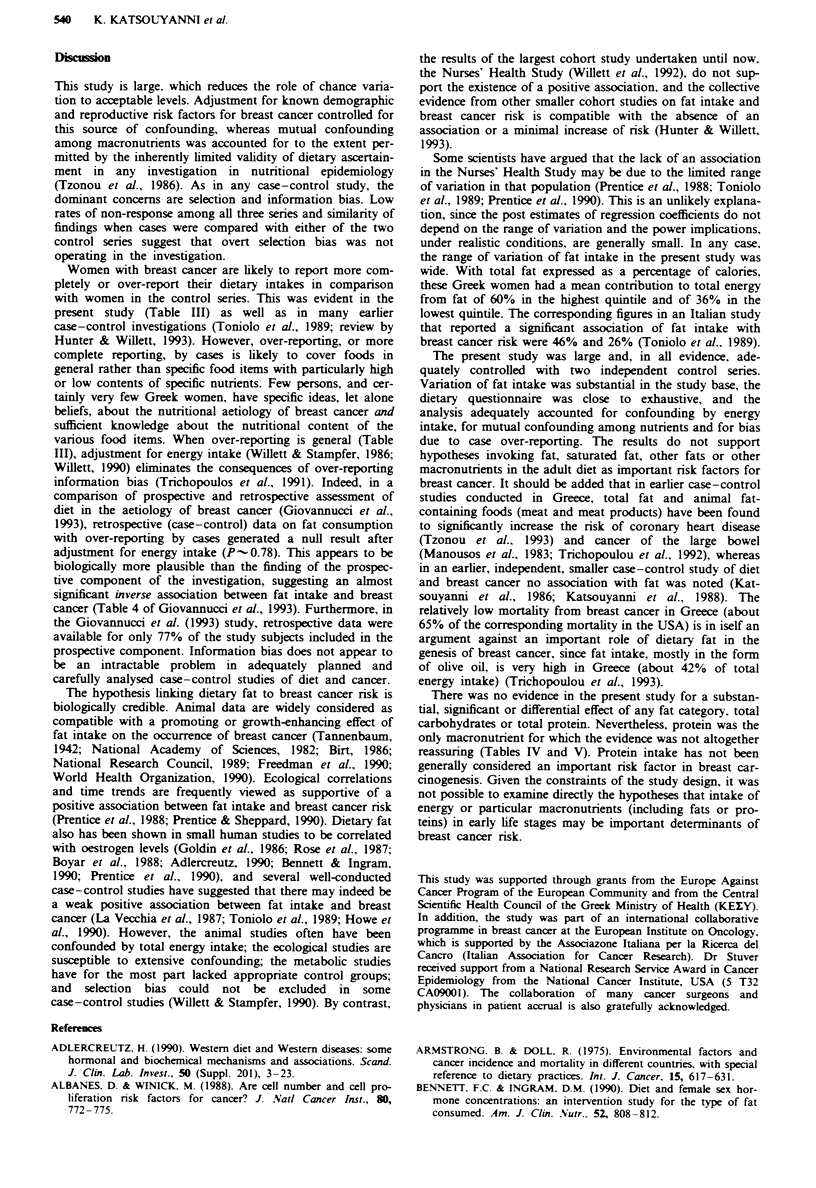

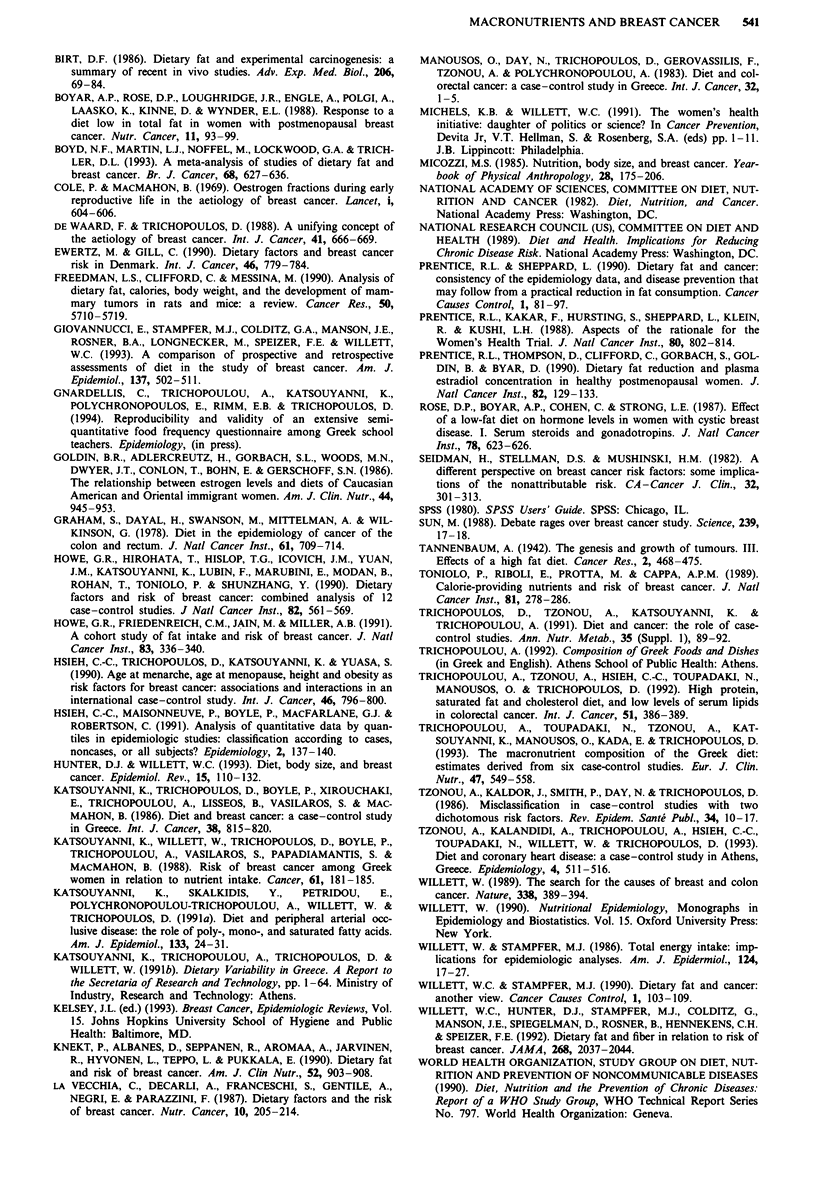


## References

[OCR_00541] Adlercreutz H. (1990). Western diet and Western diseases: some hormonal and biochemical mechanisms and associations.. Scand J Clin Lab Invest Suppl.

[OCR_00546] Albanes D., Winick M. (1988). Are cell number and cell proliferation risk factors for cancer?. J Natl Cancer Inst.

[OCR_00551] Armstrong B., Doll R. (1975). Environmental factors and cancer incidence and mortality in different countries, with special reference to dietary practices.. Int J Cancer.

[OCR_00556] Bennett F. C., Ingram D. M. (1990). Diet and female sex hormone concentrations: an intervention study for the type of fat consumed.. Am J Clin Nutr.

[OCR_00563] Birt D. F. (1986). Dietary fat and experimental carcinogenesis: a summary of recent in vivo studies.. Adv Exp Med Biol.

[OCR_00568] Boyar A. P., Rose D. P., Loughridge J. R., Engle A., Palgi A., Laakso K., Kinne D., Wynder E. L. (1988). Response to a diet low in total fat in women with postmenopausal breast cancer: a pilot study.. Nutr Cancer.

[OCR_00576] Boyd N. F., Martin L. J., Noffel M., Lockwood G. A., Trichler D. L. (1993). A meta-analysis of studies of dietary fat and breast cancer risk.. Br J Cancer.

[OCR_00579] Cole P., MacMahon B. (1969). Oestrogen fractions during early reproductive life in the aetiology of breast cancer.. Lancet.

[OCR_00588] Ewertz M., Gill C. (1990). Dietary factors and breast-cancer risk in Denmark.. Int J Cancer.

[OCR_00592] Freedman L. S., Clifford C., Messina M. (1990). Analysis of dietary fat, calories, body weight, and the development of mammary tumors in rats and mice: a review.. Cancer Res.

[OCR_00598] Giovannucci E., Stampfer M. J., Colditz G. A., Manson J. E., Rosner B. A., Longnecker M., Speizer F. E., Willett W. C. (1993). A comparison of prospective and retrospective assessments of diet in the study of breast cancer.. Am J Epidemiol.

[OCR_00612] Goldin B. R., Adlercreutz H., Gorbach S. L., Woods M. N., Dwyer J. T., Conlon T., Bohn E., Gershoff S. N. (1986). The relationship between estrogen levels and diets of Caucasian American and Oriental immigrant women.. Am J Clin Nutr.

[OCR_00621] Graham S., Dayal H., Swanson M., Mittelman A., Wilkinson G. (1978). Diet in the epidemiology of cancer of the colon and rectum.. J Natl Cancer Inst.

[OCR_00631] Howe G. R., Friedenreich C. M., Jain M., Miller A. B. (1991). A cohort study of fat intake and risk of breast cancer.. J Natl Cancer Inst.

[OCR_00624] Howe G. R., Hirohata T., Hislop T. G., Iscovich J. M., Yuan J. M., Katsouyanni K., Lubin F., Marubini E., Modan B., Rohan T. (1990). Dietary factors and risk of breast cancer: combined analysis of 12 case-control studies.. J Natl Cancer Inst.

[OCR_00642] Hsieh C. C., Maisonneuve P., Boyle P., Macfarlane G. J., Roberston C. (1991). Analysis of quantitative data by quantiles in epidemiologic studies: classification according to cases, noncases, or all subjects?. Epidemiology.

[OCR_00636] Hsieh C. C., Trichopoulos D., Katsouyanni K., Yuasa S. (1990). Age at menarche, age at menopause, height and obesity as risk factors for breast cancer: associations and interactions in an international case-control study.. Int J Cancer.

[OCR_00648] Hunter D. J., Willett W. C. (1993). Diet, body size, and breast cancer.. Epidemiol Rev.

[OCR_00664] Katsouyanni K., Skalkidis Y., Petridou E., Polychronopoulou-Trichopoulou A., Willett W., Trichopoulos D. (1991). Diet and peripheral arterial occlusive disease: the role of poly-, mono-, and saturated fatty acids.. Am J Epidemiol.

[OCR_00655] Katsouyanni K., Trichopoulos D., Boyle P., Xirouchaki E., Trichopoulou A., Lisseos B., Vasilaros S., MacMahon B. (1986). Diet and breast cancer: a case-control study in Greece.. Int J Cancer.

[OCR_00658] Katsouyanni K., Willett W., Trichopoulos D., Boyle P., Trichopoulou A., Vasilaros S., Papadiamantis J., MacMahon B. (1988). Risk of breast cancer among Greek women in relation to nutrient intake.. Cancer.

[OCR_00682] Knekt P., Albanes D., Seppänen R., Aromaa A., Järvinen R., Hyvönen L., Teppo L., Pukkala E. (1990). Dietary fat and risk of breast cancer.. Am J Clin Nutr.

[OCR_00687] La Vecchia C., Decarli A., Franceschi S., Gentile A., Negri E., Parazzini F. (1987). Dietary factors and the risk of breast cancer.. Nutr Cancer.

[OCR_00692] Manousos O., Day N. E., Trichopoulos D., Gerovassilis F., Tzonou A., Polychronopoulou A. (1983). Diet and colorectal cancer: a case-control study in Greece.. Int J Cancer.

[OCR_00723] Prentice R. L., Kakar F., Hursting S., Sheppard L., Klein R., Kushi L. H. (1988). Aspects of the rationale for the Women's Health Trial.. J Natl Cancer Inst.

[OCR_00717] Prentice R. L., Sheppard L. (1990). Dietary fat and cancer: consistency of the epidemiologic data, and disease prevention that may follow from a practical reduction in fat consumption.. Cancer Causes Control.

[OCR_00730] Prentice R., Thompson D., Clifford C., Gorbach S., Goldin B., Byar D. (1990). Dietary fat reduction and plasma estradiol concentration in healthy postmenopausal women. The Women's Health Trial Study Group.. J Natl Cancer Inst.

[OCR_00734] Rose D. P., Boyar A. P., Cohen C., Strong L. E. (1987). Effect of a low-fat diet on hormone levels in women with cystic breast disease. I. Serum steroids and gonadotropins.. J Natl Cancer Inst.

[OCR_00740] Seidman H., Stellman S. D., Mushinski M. H. (1982). A different perspective on breast cancer risk factors: some implications of the nonattributable risk.. CA Cancer J Clin.

[OCR_00748] Sun M. (1988). Debate rages over breast cancer study.. Science.

[OCR_00756] Toniolo P., Riboli E., Protta F., Charrel M., Cappa A. P. (1989). Calorie-providing nutrients and risk of breast cancer.. J Natl Cancer Inst.

[OCR_00761] Trichopoulos D., Tzonou A., Katsouyanni K., Trichopoulou A. (1991). Diet and cancer: the role of case-control studies.. Ann Nutr Metab.

[OCR_00777] Trichopoulou A., Toupadaki N., Tzonou A., Katsouyanni K., Manousos O., Kada E., Trichopoulos D. (1993). The macronutrient composition of the Greek diet: estimates derived from six case-control studies.. Eur J Clin Nutr.

[OCR_00769] Trichopoulou A., Tzonou A., Hsieh C. C., Toupadaki N., Manousos O., Trichopoulos D. (1992). High protein, saturated fat and cholesterol diet, and low levels of serum lipids in colorectal cancer.. Int J Cancer.

[OCR_00786] Tzonou A., Kalandidi A., Trichopoulou A., Hsieh C. C., Toupadaki N., Willett W., Trichopoulos D. (1993). Diet and coronary heart disease: a case-control study in Athens, Greece.. Epidemiology.

[OCR_00782] Tzonou A., Kaldor J., Smith P. G., Day N. E., Trichopoulos D. (1986). Misclassification in case-control studies with two dichotomous risk factors.. Rev Epidemiol Sante Publique.

[OCR_00810] Willett W. C., Hunter D. J., Stampfer M. J., Colditz G., Manson J. E., Spiegelman D., Rosner B., Hennekens C. H., Speizer F. E. (1992). Dietary fat and fiber in relation to risk of breast cancer. An 8-year follow-up.. JAMA.

[OCR_00801] Willett W., Stampfer M. J. (1986). Total energy intake: implications for epidemiologic analyses.. Am J Epidemiol.

[OCR_00792] Willett W. (1989). The search for the causes of breast and colon cancer.. Nature.

[OCR_00584] de Waard F., Trichopoulos D. (1988). A unifying concept of the aetiology of breast cancer.. Int J Cancer.

